# Molecular and Functional Characterization of a c-type lysozyme from the Asian Corn Borer, *Ostrinia furnacalis*

**DOI:** 10.1673/031.009.1701

**Published:** 2009-05-08

**Authors:** Wen-Xian Wang, Yi-Peng Wang, Xiao-Juan Deng, Xiang-Li Dang, Jin-Huan Tian, Hui-Yu Yi, Yi-Feng Li, Xiao-Fang He, Yang Cao, Qing-You Xia, Ren Lai, Shuo-Yang Wen

**Affiliations:** ^1^ Department of Entomology, College of Natural Resource and Environment, South China Agricultural University, Guangzhou, 510642, P. R. China; ^2^ Kunming Institute of Zoology, Chinese Academy of Sciences. Kunming, 650223, P. R. China; ^3^ Department of Sericulture Science, College of Animal Science, South China Agricultural University, Guangzhou, 510642, P. R. China; ^4^ Zhejiang Institute of Subtropical Crops, Wenzhou, 325005, P. R. China; ^5^ Department of Material and Engineering, Jinan University, Guangzhou, 510632, P.R. China; ^6^ Key Sericultural Laboratory of Agriculture Ministry, College of Sericulture and Biotechnology, Southwest University, Chongqing, 400716, P. R. China; ^7^ Graduate School of Chinese Academy Sciences, Beijing, 100091, P. R. China

**Keywords:** OstrinLysC, antibacterial activity, expression pattern, charged residue, hydrophobic residue

## Abstract

Some lepidopteran lysozymes have been reported to display activity against Gram-positive and Gram-negative bacteria, in contrast to most lysozymes that are active only against Gram-positive bacteria. OstrinLysC, a c-type lysozyme, was purified from the Asian corn borer, *Ostrinia furnacalis* Guenée (Lepidoptera: Pyralidae), and shows activity against Gram-positive and Gram-negative bacteria. The NH2-terminal amino acid sequence was determined by Edman degradation and used in a homology cloning strategy. The gene coding for OstrinLysC contains three exons and two introns. The expression profile of the *OstrinlysC* gene was examined by quantitative real-time PCR. Following injection of the larvae with bacteria, the *OstrinlysC* gene is strongly up-regulated in immune tissues. Transcripts were also detected in gut tissue. After feeding the larvae with bacteria, *OstrinlysC* transcripts increased in immune tissues. A very low level of transcript abundance was also detected in gut tissue. These results suggested that the *OstrinlysC* gene is involved in immune responses. The three dimensional structure of OstrinLysC was predicted. Based on comparison of the 3-D structure of OstrinLysC with that of silkworm lysozyme and chicken lysozyme, we hypothesize that the positive charge-rich surface and the short loop-2, which is close to the cluster of hydrophobic residues, may play important roles in the interaction with the outer membrane of Gram-negative bacterial cell walls.

## Introduction

Innate immunity allows insects to protect themselves against a wide range of microbial pathogens that cause infection. Insect innate immune systems comprise cellular and humoral defense responses (Boman et al. 1987; Hoffmann 1995; Hoffmann et al. 1996; Bulet et al. 1999; Lavine et al. 2002). The humoral defense response takes effect by over-expressing an array of potent antimicrobial proteins and peptides at the time of pathogenic infection (Bulet et al. 1999; Hoffmann and Reichhart 2002). Among the large number of inducible antimicrobial proteins and peptides, lysozyme is the most ubiquitous antibacterial factor and is widely distributed in vertebrate and invertebrate animals.

Since the first insect lysozyme was recognized in honeybees (Mohrig et al. 1968), more than fifty lysozyme genes have been identified from several insect orders, including Diptera, Lepidoptera, Orthoptera (Hultmark 1996), Isoptera (Fujita et al. 2002) and Hemiptera (Araujo et al. 2006). In Dipteran species, the lysozyme genes cluster as a multi-gene family. For example, in the African malaria mosquito *Anopheles gambiae,* eight chicken type (c-type) lysozyme gene members (*Lys c1-8*) form the c-type lysozyme gene family (Li et al. 2005; Waterhouse et al. 2007). In another mosquito *Aedes aegypti,* there are six c-type lysozyme genes (*Lys-A, Lys-B, Lys-C, Lys-D, Lys-E* and *Lys-G*) in the genome (Gao et al. 2000!; Ursic Bedoya et al. 2005; Li et al. 2005; Waterhouse et al. 2007). *Ae. aegypti Lys-A* is constitutively expressed and up-regulated upon immune challenge and blood feeding in adult mosquitoes while *Ae. aegypti Lys-E* is expressed at low levels in adults after immune challenge (Ursic Bedoya et al. 2005). In *Drosophila melanogaster,* eighteen lysozyme genes have been identified. Thirteen (*LysA, LysB, LysC, LysD, LysE, LysP, LysS, LysX,* CG7798, CG30062, CG11159, CG16756 and CG16799) encode c-type lysozymes, and five encode invertebrate type (i-type) lysozymes (*D. melanogaster* 1–3, CG6426 and CG6429) (Daffre et al. 1994; Regal et al. 1998; Bachali et al. 2002; Waterhouse et al. 2007; GenBank accession number: NM137319 and NM137320). c-type and i-type lysozymes have a common ancestor domain but diverged in their NH2-terminal and COOH-terminal domains. *D. melanogaster* is the first metazoan which is reported to have both c-type and i-type proteins (Bachali et al. 2002). The dipteran lysozyme genes have distinctively temporal and spatial patterns of expression, and have evolved into two functions: 1) digestive, breaking down ingested bacteria in the gut and, 2) defensive, responding against pathogens that enter the haemocoel (Regel et al. 1998; Ursic Bedoya et al. 2005). In contrast, there is only one copy of a functional c-type lysozyme gene in the lepidopteran genome and the gene product plays a very important role in the defensive function. (Sun et al. 1991; Mulnix et al. 1994; Jain et al. 2001; Yu et al. 2002; Matsuura et al. 2002; Bachali et al. 2002). Recently, some lysozyme-like proteins were identified in *Bombyx mori* and *Antheraea mylitta* that display activity against Gram-positive and Gram-negative bacteria. Such lysozyme-like proteins share 50–60% amino acid similarity with c-type insect lysozymes but lack catalytic activity of peptidoglycan hydrolysis (Gandhe et al. 2007).

So far, fifteen c-type lysozymes have been identified in lepidopteran insects. Among them, cDNA of ten lysozyme genes has been sequenced from *Hyalophora cecropia* (Engstrom et al. 1985), *Manduca sexta* (Mulnix et al. 1994), *Bombyx mori* (Lee et al. 1995), *Trichoplusia ni* (Kang et al. 1996), *Hyphantria cunea* (Park et al. 1997), *Heliothis virescens* (Shelby et al. 1998), *Samia cynthia* (Fujimoto et al. 2001), *Spodoptera exigua* (Bae et al. 2003), *Helicoverpa zea* (Liu et al. 2004) and *Pseudoplusia includens* (Lavine et al. 2005). The structure and the amino acid sequence of a lysozyme from *A. mylitta* have been characterized (Jain et al. 2001). A comparison of the characteristics of lysozyme from *Galleria mellonella* and *Agrius convolvuli* has been conducted (Yu et al. 2002). Moreover, there are two unpublished lysozyme gene sequences from *Artogeia rapae* and *Antheraea pernyi* in GenBank.

Lysozymes are defined by their enzymatic hydrolysis of the β″1, 4-glycosidic linkage between *N*-acetylmuramic acid and *N*-acetylglucosamine of the peptidoglycan layer in the bacterial cell walls (Jolles and Jolles 1984; Prager and Jolles 1996). This mechanism is basically directed against certain Gram-positive bacteria and to lesser extent against Gram-negative bacteria (Ibrahim et al. 2002). Among known lysozymes, three lysozymes from lepidopteran insects, *G. mellonella, B. mori* and *A. convolvuli,* have been reported to display weak activity against Gram-negative bacteria (Yu et al. 2002; Abraham et al. 1995). In this study, a c-type lysozyme, OstrinLysC, was purified and characterized from the Asian corn borer, *Ostrinia furnacalis* Guenée (Lepidoptera: Pyralidae). It showed activity against two Gram-positive bacterial species and two Gram-negative bacterial species. Potential factors influencing the interaction of lysozyme with the outer membrane of Gram-negative bacterial cell wall is discussed based on a detailed comparison of three dimensional structures of silkworm lysozyme (*Bm*LZ), chicken lysozyme (HEWLZ) and the predicted model of OstrinLysC.

## Materials and Methods

### Insects, bacteria

*O. furnacalis* were reared on a semi-artificial diet at 26°C and 80% RH with a photoperiod of 16:8 L:D (Song et al. 1999). The bacteria, *Escherichia coli* K12D31, and *Staphylococcus aureus* were cultured in Luria-Bertani medium (1% tryptone, 0.5% yeast extract, 1% NaCl w/v; adjusted pH 7.0–7.2 with 10 mol/L NaOH). *Pseudomonas aeruginosa, Bacillus thuringiensis* var. *galleriae, Ralstonia solanacearum* and *Bacillus subtilis* were cultured in nutrient agar broth (1% tryptone, 0.5% beef broth and 0.5% NaCl w/v, pH7.0–7.2). All bacteria were obtained from the Guangzhou Institute of Microbiology (Guangzhou, China).

### Insect immunization, haemolymph collection and purification of lysozyme

Fifth-instar *O. furnacalis* larvae were injected with 2 µl of the bacterial mixture of *E. coli* K12D31 and *S. aureus* (about 2×105 cells of each bacterial species suspended in 2 µl PBS) and kept at 26°C for 24 hours. Infected larvae were then chilled on ice. The hemolymph was collected into the extracting buffer (containing 1 µg/ml of aprotinin and 10 µM phenylthiourea) and centrifuged at 12,000 rpm for 10 min at 4°C. The supernatant was stored at -40°C until further purification.

The supernatant was equilibrated with ammonium acetate-acetic acid buffer (50 mM NH4OAc, pH5.0) and filtered through a membrane filter (0.22 µm pore size, Millipore, www.waters.com). The filtrate was applied to a CM-Sepharose Fast Flow column (Amersham Pharmacia Biotech, www.apbiotech.com) and eluted with a linear gradient of 0.05–1 M NH4OAc in the same buffer at a flow rate of 1.5 ml/min. The antibacterial fractions were pooled and lyophilized in the Freeze Dry System/Freezone® 4.5 (Labconco, www.labconco.com), then resuspended in 0.1% trifluoroacetic acid buffer and applied to a Resource RPC 3 ml column (6.4 × 100 mm, Amersham Pharmacia Biotech) in the ÄKTA FPLC system (Amersham Pharmacia Biotech). The antibacterial substances were eluted with a linear gradient of 0–80% acetonitrile in 0.1% trifluoroacetic acid acidified water. The flow rate was 1 ml/min. The antibacterial fraction was applied to the Hypersil ODS C18 column (4.6 × 100 mm, Dalian Elite, www.elitehplc.com) in the Agilent 1100 serial HPLC system (Agilent Technologies, www.agilent.com) with a linear gradient of 30%–40% acetonitrile in acidified water. The flow rate was 0.7 ml/min. The elution pattern of antibacterial substances in each purification step was monitored by measuring the absorbance at 280 nm.

### Antibacterial activity assay

In each purification step, antibacterial activity against *E. coli* and *S. aureus* was analyzed by a radial diffusion assay on agar plates seeded with bacteria (Lambert et al., 1989) with minor modification. Luria-Bertani agar (1.5%) or nutrient agar (1.5%), containing 60 µl of a suspension of bacteria (grown until the OD600 reached 0.5), were poured into Petri dishes. Holes (3 mm in diameter) were punched in the agar. Test samples (10 µl at 100 µg/ml) were loaded into each hole. The plates were incubated at 37°C for 18 hours, and then the radius of the clear zone around each hole was measured. The bacterial species *E. coli, S. aureus, P. aeruginosa, B. thuringiensis* var. *galleriae, R. solaanacearum* and *B. subtilis,* were employed for investigating the antibacterial spectrum of the purified antibacterial fraction. The Luria-Bertani agar was used for *E. coli* and *S. aureus* and nutrient agar was for *P. aeruginosa, B. thuringiensis* var. *galleriae, R. solanacearum* and *B. subtilis.*

### Tricine SDS-PAGE and amino acid sequencing

To determine the purity and the molecular weight of the purified antibacterial fraction, Tricine-SDS polyacrylamide gel electrophoresis was carried out using a 4% stacking gel and 16.5% separating polyacrylamide gel as described by Schägger and von Jagow (1987). After electrophoresis, the gel was stained in Coomassie brilliant blue R-250 (Sigma, www.sigmaaldrich.com). The antibacterial protein from the final step of purification was sequenced for N-terminal amino acid residues on an ABI 476A gas-phase automatic sequencer (Applied Biosystems, www.appliedbiosystems.com) using the Edman degradation method.

### cDNA cloning and nucleotide sequencing

Total RNA was extracted from immunized fifth-instar larvae of *O. furnacalis* using TRIzol (Invitrogen, www.invitrogen.com) based on the user manual. Genomic DNA was extracted using Genomic DNA extraction Kit (Tiangen, www.tiangen.com). cDNA was synthesized from 0.5 µg total RNA using a SMART™ cDNA library construction kit (Clontech, ww.clontech.com) according to the user manual. Briefly, first strand cDNA was synthesized with CDS primer and SMART™ oligonucleotide (Table 1). Second strand cDNA was amplified with 5′ PCR primer and 3′ PCR primer (Table 1). RT-PCR was carried out with the 3′ PCR primer and the degenerate primer (Table 1) based on the N-terminal amino acid sequence (Figure 2: from I8 to R13). Finally, 5′-RACE was carried out using 5′ PCR primer and lysR1 (Table 1). Two primer sets, lysF1/lysR1 and lysF2/lysR2 (Table 1), were designed to amplify introns according to the alignment of the sequences of cDNA of *OstrinLysC* gene and the genome sequences of lepidopteran lysozyme genes from GenBank. All DNA fragments were purified using Agarose Gel DNA Purification Kit (Takara) and Cloned using pGEM-T Easy Vector System (Promega, www.promega.com). Nucleotide sequences were determined by 3730 DNA analyzer (Applied Biosystems).

### Quantitative real time PCR (qRT-PCR)

Fifth instar larvae were infected in two ways: injecting or feeding with bacteria. Fifth instar larvae were injected with bacteria as described above. After 24 hours, the larvae were dissected for tissue collection. For oral feeding, the molting fifth instar larvae were reared on semi-artificial diet mixed with bacteria (2×107 bacteria cells/mg diet) for 24 hours. Immunized fifth instar larvae were chilled on ice. Haemolymph was collected into the extracting buffer (containing 1 µg/ml of aprotinin and 10 µM phenylthiourea) and then the larvae were dissected on ice for collecting the fat body and gut. Haemolymph was centrifuged at 6,000 g for 5 min at 4°C to collect the haemocytes. Haemocytes, fat body and gut were stored in liquid nitrogen until total RNA extraction. Total RNA of immunized tissues was extracted using TRIzol (Invitrogen) based on the user manual. Quantiative real-time PCR was performed by SYBR PrimeScript RT-PCR kit (Perfect Real Time) (Takara, www.takarabio.co.jp). Briefly, cDNA was synthesized from 0.5µg total RNA with oligo dT primer according to the manufacturer's protocol. Reaction mixture (25 µl) included 12.5 µl SYBR Green Real-time PCR Master Mix, 0.2 µmol/L of forward primer (lysF, Table 1), 0.2 µmol/L of reverse primer (lysR, Table 1), 0.5 µl Rox preference dye (50 ×) and 0.5 µl cDNA. A ribosomal protein gene (rpL8) was used as an internal control. Primer set RP-F/RP-R (Table 1) was used to amplify the rpL8. The qRT-PCR was run with the 96-well plate on ABI Prism® 7300 Real Time PCR system (Applied Biosystems), followed the program of 95° C for 10s, 40 cycles of 95° C for 5s, 55° C for 15s, and 72° C for 31s. The template amount of lysozyme transcripts was normalized against rpL-8.

**Table 1.**
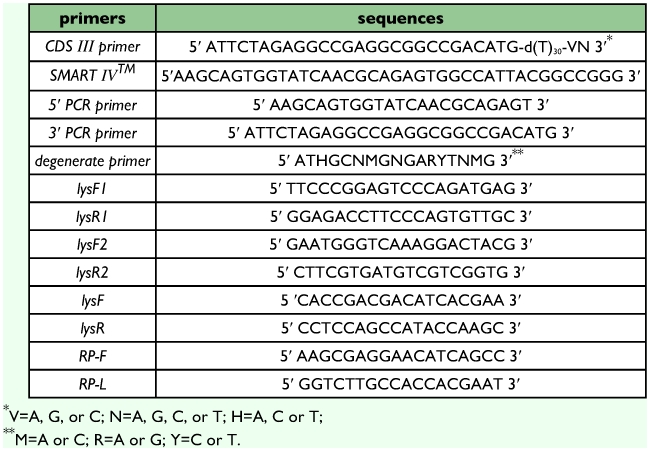
Nuclear sequences of primers

### Data analysis

Protein homology search for the NH2-terminal sequence and nucleotide homology search for the DNA sequence were performed by Blast on the NCBI website (http://www.ncbi.nlm.nih.gov/BLAST). Sequence editing, alignment, and the phylogenetic analysis were performed using MEGA 4.1 (http://www.megasoftware.net). The molecular weight and isoelectric point of protein were estimated using ProtParam tool (http://www.expasy.org/tools/protparam.html). The signal peptide was predicted by SignalP (http://www.cbs.dtu.dk/services/SignalP/). The three dimensional structure of OstrinLysC was predicted based on the known structure of silkworm lysozyme (Matsuura et al. 2002) and chicken lysozyme (Wilson et al. 1992) using the Swiss-PdbViewer v3.7 (http://www.expasy.org/spdbv/).

## Results

### Purification and the antibacterial activity of OstrinLysC

**Figure 1.**
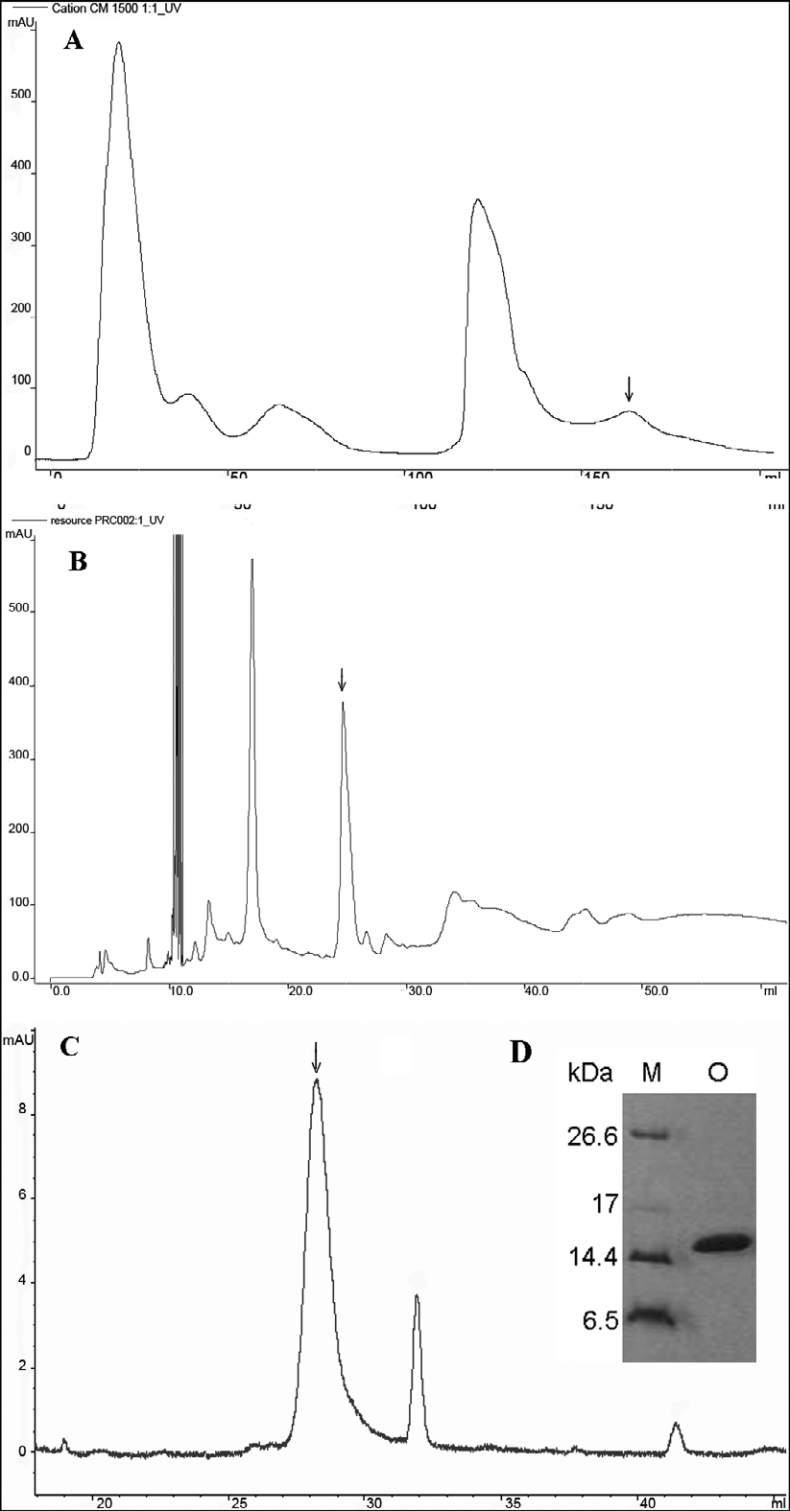
Purification of OstrinLysC from the hemolymph of immunized *Ostrinia furnacalis.* Arrows indicated target fractions with antimicrobial activity. A. By cation exchange chromatography with CM-Sepharose column. B. Target fraction in A was further purified by RP-FPLC with the Resource RPC 3ml column (6.4 × 100 mm). C. Target fraction in B was purified by RP-HPLC with Hypersil ODS C18 column (4.6 × 250 mm), this antimicrobial activity fraction was used for amino acid sequencing. D. Target fraction in C on the analytical tricine SDS-PAGE gel (M: molecular weight marker. O: Target fraction in C).

The hemolymph of immunized fifth-instar *O. furnacalis* displayed high antimicrobial activity against *E. coli* K12D31 and *S. aureus* and reached maximum activity 24 hours after immunization (data not shown). The collected hemolymph was fractionated by a weak cation exchanger (CM sepharose) and an antibacterial fraction was obtained (Figure 1A). This fraction was subsequently purified using a reverse phase column (Resource RPC 3ml) in a FPLC system. A fraction displaying antibacterial activity (Figure 1B) was further purified using a reverse phase column (Hypersil ODS C18) in an HPLC system. A peak (Figure 1C) was found to be strongly active against *S. aureus* and weakly active against *E. coli.* The molecular weight of this antibacterial peak fraction on the Tricine-SDS PAGE was about 15 kDa (Figure 1D). The N-terminal amino acid residues of this antibacterial fraction were sequenced by Edman degradation. Fifteen amino acid residues were obtained with the following sequence: KILKR*DIARELRSQ (* denotes unknown residue). This sequence was 42.9% identical to the lysozyme of *A. mylitta.* These results suggest that this antibacterial protein might be a lysozyme. This protein is termed OstrinLysC.

**Figure 2.**
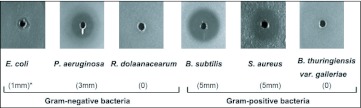
Antibacterial activities of OstrinLysC against Gram-positive and Gram-negative bacteria. *: semidiameter of clear zone. OstrinLysC displayed antibacterial activity against two Gram-positive bacteria species and two Gram-negative bacteria species: 5 mm of semidiameter clear zone against *B*. *subtilis* and *S. aureus.* 3 mm of semidiameter clear zone against *P*. *aeruginosa* and 1 mm of semidiameter clear zone against *E*. *coli.* 10 µl of OstrinLysC (100 µg/mL) was used for assay.

The antibacterial spectrum of OstrinLysC was assayed with six bacterial species. As shown in Figure 2, OstrinLysC displayed strong antibacterial activity against two Gram-positive bacteria, *B. subtilis* (ca, 5mm radius of clear zone) and *S. aureus* (ca. 5 mm radius of clear zone). OstrinLysC also inhibited two Gram-negative bacterial speciess, *E. coli* (ca. 1 mm radius of clear zone) and *P. aeruginosa* (ca. 3 mm radius of clear zone). However, OstrinLysC didn't display antibacterial activity against another Gram-negative bacterium, *R. solanacearum,* or the Gram-positive bacterium, *B. thuringiensis* var. *galleriae.* These data show that OstrinLysC not only has activity against Gram-positive bacteria, but also Gram-negative bacteria.

### Characterization of the OstrinLysC gene

**Figure 3.**
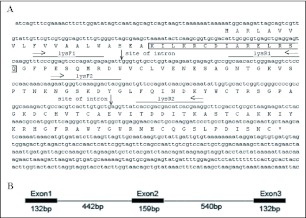
cDNA sequence of the *OstrinLysC* gene (GenBank accession number: EF120625) and the deduced amino acid sequences of OstrinLysC. The cDNA sequence is indicated in the lowercase, the amino acid sequence is in the uppercase. Residues that were sequenced using the Edman degradation are indicated with the shaded box. Residues upstream of the shaded box are sequences of the signal peptide. Sequences under the thick line are two primer sets (lysF1/lysR1 and lysF2/lysR2) used for PCR, the directions of primer are indicated by horizontal arrows. Vertical arrows show the site of two introns. The putative polyadenylation signal is indicated by broken line.

The full length cDNA sequence was obtained by combining sequences of the 3′-RACE and 5′-RACE. It encodes a 140 amino acid residue peptide that contains a 20 amino acid signal peptide and a 120 amino acid mature peptide. Two introns were obtained from the genome using the primers based on the full-length cDNA sequence (Figure 3 A). The exon-intron structure of *OstrinLysC* gene is indicated in Figure 3 B. There are three exons and two introns in the *OstrinLysC* gene. The sizes of the exons are 132bp, 159bp and 132bp respectively, and the introns are 442bp and 540bp respectively. The sequence of *OstrinLysC* gene was submitted to GenBank (EF120625).

The molecular weight and isoelectric point of OstrinLysC were predicted to be 13563.3 Daltons and 8.95 respectively. The DNA and amino acid sequence of OstrinLysC is highly homologous with c-type lysozymes of other insects and chicken suggesting that it belongs to the c-type lysozyme family.

### Sequence alignment and phylogenetic analysis

**Figure 4.**
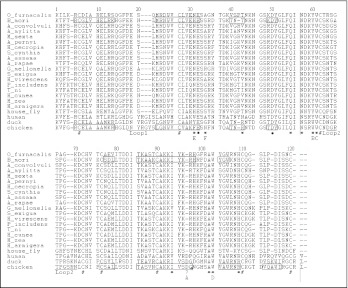
Sequence alignment of OstrinLysC with the chicken, duck, human, housefly and lepodipteran lysozymes. Gaps are indicated by “-”, eight conserved cysteines are indicated by “#”,“-” indicates two catalytic sites in case of lysozymes of *B. mori* and chicken, “?” indicates another catalytic site in case of chicken lysozyme, this site may be inhabited by the salt bridge between Asp 70 and Arg 96 in *B. mori*. The residues corresponding to the active site in the case of tasar silkworm lysozyme are denoted with “*”. A-F indicates substrate-binding sites in the case of chicken lysozyme. Residues forming secondary structures of lysozymes in *B. mori* and chicken are indicated in boxes. Two loops in the secondary structures are underlined.

The amino acid sequence of OstrinLysC was aligned with seventeen lepidopteran lysozymes, as well as the lysozymes from the housefly, human, duck and chicken. As shown in Figure 4 OstrinLysC possesses 8 conserved cysteine residues (Cys6, Cys27, Cys62, Cys72, Cys76, Cys90, Cys110, Cys120) and two catalytic sites of glutamic acid (Glu32) and aspartic acid (Asp50), which are fundamental for the three dimensional structure and the biological activity of the c-type lysozyme. These residues are conserved in c-type lysozymes. Examination of twelve active sites of tasar silkworm lysozyme (TSWAB) (Jain et al. 2001) and six substrate-binding sites of chicken lysozyme (Kumagai et al. 1993) determined that most of them are common in the OstrinLysC and other lepidopteran lysozymes (Figure 4). Among these residues, Qln55, Asn57, Trp61 (substrate-binding site C), Ala102 and Trp103 (substrate-binding site D) are present in all c-type lysozymes. Tyr60 (substrate-binding site B) and His are present in all lepidopteran lysozymes and housefly lysozyme. Ile94 is identical in all lysozymes except the lysozyme of *G. mellonella* and human lysozyme. Arg97 is identical in all lepidopteran lysozymes. Asn31 (substrate-binding site E) is identical in all lepidopteran lysozymes except silkworm lysozyme. Ala34 (substrate-binding site F) is identical in OstrinLysC and lysozymes of *A. mylitta* and *H. virescens.* Thr43 is identical in OstrinLysC, silkworm and housefly lysozymes. This comparison revealed that 45.5% of the active site amino acids are conserved in c-type lysozymes and 63.6% active site amino acids are conserved in lepidopteran lysozymes. The substrate-binding sites, C and D, are conserved in all c-type lysozymes. Substrate binding site B is tyrosine in lepidopteran, housefly, and human lysozymes, but is tryptophan in duck and chicken lysozymes. Substrate binding site E is asparagine in lepidopteran lysozymes except that of silkworm lysozyme; it is histidine in silkworm and housefly lysozymes and varies in human, duck and chicken lysozymes. Substrate binding site A is absent in lepidopteran lysozymes and housefly lysozyme while it is aspartic acid in lysozyme of human, duck and chicken. Substrate binding site F varies across c-type lysozymes. This analysis revealed that most substrate-binding sites are conserved in lepidopteran lysozymes except substrate-binding site F. Thus, the antimicrobial activity might be more similar between lepidopteran lysozymes than other lysozymes.

Additional structural features support this argument. Of two loops found in the secondary structure of lysozymes, the size of loop-1 is identical in lepidopteran lysozymes and housefly lysozyme (4 residues) while it is longer in human, duck and chicken lysozymes (7 residues). The size of loop-2 is conserved among lepidopteran lysozymes (7 residues) while it is longer in housefly, human, duck and chicken lysozymes (9 residues).

**Figure 5.**
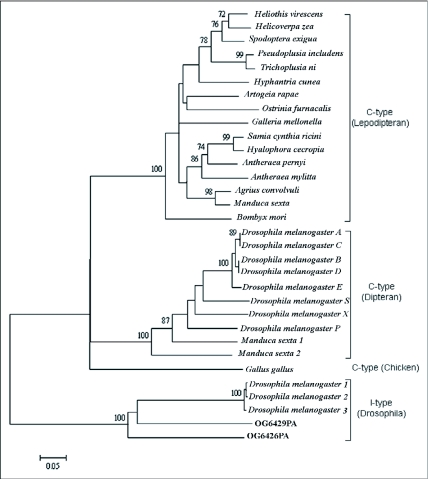
NJ tree of Lepidopteran lysozymes and a subset of dipteran lysozymes. Sequences of mature peptide of c-type lysozymes were analyzed by Mega3.1. using the i-type lysozymes as the outgroup (Bootstrap test: 10050 replications; Amino acid substitution model: p-distance; Gaps were treated by pairwise delection), Numbers at the nodes indicated the bootstrap proportions (only over 70% were showed). The 0.05 scale is for the branch length which indicated relative p-distance.

The phylogenetic relationship of c-type lysozymes from Lepidoptera, Diptera and chicken was analyzed by the neighbor joining method using i-type lysozymes as out-group. As Figure 5 shows, the dipteran and depidopteran lysozymes separate into two branches. The OstrinLysC is a close relative with the lysozyme of *A. rapae* in the depidopteran branch.

### Expression Pattern of OstrinLysC Gene

**Figure 6.**
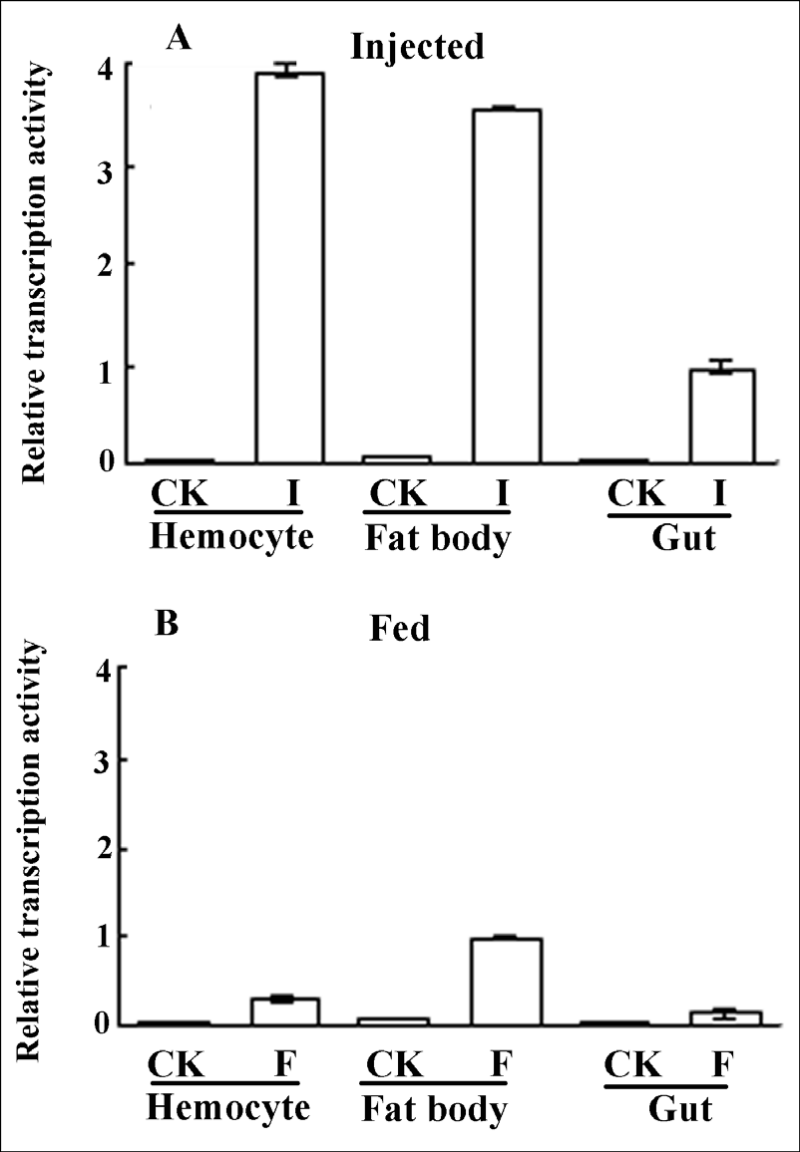
qRT-PCR analysis on the expression of *OstrinLysC* gene in immunized tissues of fifth instar larvae of *Ostrinia furnacalis.* A: Relative transcription activity of *OstrinLysC* gene in immunized tissues after injecting five instar larvae with bacteria. The *OstrinLysC* gene was up-regulated strongly in the hamocyte and fat body. The transcripts were also detectable in gut. B: Relative transcription activity of *OstrinLysC* gene in Immunized tissues after feeding five instar larvae with bacteria. The *OstrinLysC* gene was up-regulated mainly in fat body and hamocyte. Very low activity of transcription is detected in gut.

The expression pattern of *OstrinLysC* gene was screened in immune tissues and digestive tissue by qRT-PCR. Transcript abundance of *OstrinLysC* increased strongly in the fat bodies and hemocytes of fifth instar larvae after injection with bacteria (Figure 6). The transcript abundance increased in the gut of larvae after injection with bacteria. After feeding the fifth instar larvae with bacteria, the *OstrinLysC* transcripts increased in hemocytes and fat body. A low level of transcripts was detectable in the gut. Such results suggested that expression of the *OstrinLysC* gene is inducible in the immune tissues and in the gut.

### Prediction of three-dimensional structure of OstrinLysC

**Figure 7.**
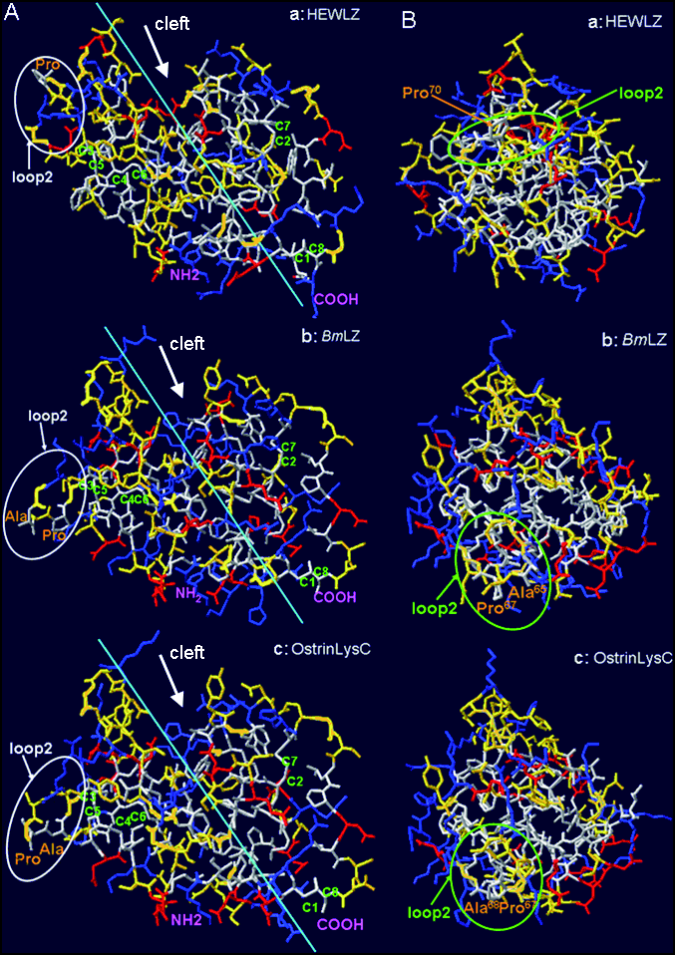
Comparison of the three dimension structure of lysozymes from the chicken (HEWLZ), the silkworm (*Bm*LZ) and the *Ostrinia furnacalis* (OstrinLysC). The 3-D structure of OstrinLysC was predicted Based on the 3-D structure of HEWLZ (PDB code: IHEL, Wilson et al. 1992) and *Bm*LZ (PDB code: IGD6, Matsuura et al. 2002) using the Swiss PdbViewer 3.7 (http://www.expasy.org/spdbv/). Positively and negatively charged residues are shown in blue and red; Hydrophobic and hydrophilic residues are indicated in white and yellow. A: The general 3-D structure showing a conservative cleft of cleavage sites (arrow pointed), secondary structure (Almost α-helixes distributed above the line, all β″-sheets distributed below the line), the loop-2 (white circled) and four internal disulfide bridges (green letters: C1-C8, C2-C7, C3-C5, C4-C6). B: The 3-D structure in A was anticlockwise 90° rotated to show the distribution of positive charged residues. A positive charged-rich side was found in the surface of *Bm*LZ and OstrinLysC (left side) but in the HEWLZ. The location of Loop 2 (green circled) is upper in the HEWLZ and down in the *Bm*LZ and OstrinLysC.

The three dimensional structure of OstrinLysC was predicted using the Swiss-PdbViewer v3.7 based on the known three dimensional structure of silkworm lysozyme (1gd6A), (Matsuura et al. 2002) and chicken lysozyme (1HEL) (Wilson et al. 1992) as shown in Figure 7-A. Some common characters are found based on the comparison of three lysozymes: the conformation of the main secondary structures including α-helices, β″-sheets, and a deep cleft with two catalytic site residues. However, two remarkable differences were found between lepidopteran and chicken lysozymes. First, the location of loop-2 is significantly different between lepidopteran lysozyme and HEWLZ. The loop-2 of lepidopteran lysozymes is in the middle left of the structure (Figure 7A-b, c), while the loop-2 of HEWLZ is in the upper left of the structure (Figure 7A-a). Probably, the two-residue deletion in loop-2 of lepidopteran lysozymes (Figure 4) leads the loop-2 to be moved. Second, there is a positive-charge rich area in OstrinLysC and BmLZ (Figure 7B-b, c), but not in HEWLZ (Figure 7B-a).

## Discussion

In this study OstrinLysC was purified and identified from immunized larval hemolymph of *O. furnacalis.* Based on a comparison of residues of the known c-type lysozymes (Figure 4), OstrinLysC was found to have similar molecular characters to the c-type lysozymes: eight cysteine residues for forming the disulfide bridges, two catalyzing sites (Glu32 and Asp50), and five substrate-binding sites (E: Asn31, F: Ala34, B: Tyr60, C: Trp61 and D: Trp103) for hydrolyzing the peptidoglycan. Among these substrate-binding sites, only substrate-binding site F varies among lepidopteran lysozymes. The substrate-binding site A of chicken lysozyme is missing in lepidopteran lysozymes. This residue profile suggests that the muramidase activity of OstrinLysC might be very similar to that of other known lepidopteran lysozymes. In the antibacterial activity bioassay, three Gram-positive bacterial species and three Gram-negative bacterial species were used as the test bacteria. Among these six species, OstrinLysC showed strong activity against two species of Gram-positive bacteria and a Gram-negative bacterium. Weak activity against Gram-negative bacterium *E. coli* was also detectable (Figure 2). This is the fourth lepidopteran lysozyme that is reported to display activity against Gram-negative bacteria.

The spatial and temporal expression of lysozyme genes varies among dipteran and lepidopteran insects. The transcriptional profile of the *OstrinLysC* gene was examined in this study. The expression of *OstrinLysC* was up-regulated mainly in immune tissues after challenge. A lower level of relative transcription activity was also detected in the gut of larvae after injecting or oral feeding with bacteria (Figure 6). This profile is similar to that seen in the lepidopteran *S. cynthia ricini* (Fujimoto et al. 2001). We suggest that lysozyme in lepidopteran species might play a more dominant role in immune response rather than in the digestive function. The dipteran c-type lysozyme multi-gene family plays a very important role in the digestive system (Daffre et al. 1994; Regel et al. 1998; Bachali et al. 2002; Bedoya et al. 2005; Li et al. 2005; Araujo et al. 2006). In the *An. gambiae* and *Ae. aegypti* genome, some lysozymes are responsive to immune challenges and some may have digestive functions (Li et al. 2005; Bedoya et al. 2005). Some *Drosophila* lysozymes digest the bacteria in the guts (Daffre et al. 1994; Regel et al., 1998). Several lysozyme genes in *Drosophila* are not induced in response to bacteria and virus (De Gregorio et al., 2001; Roxstrom-Lindquist et al. 2004), but some are downregulated in response to fungus, and others are up-regulated in response to a parasite (*Octosporea*) infection (Roxstrom-Lindquist et al. 2004). It is obvious that the main function of lysozyme is very different between lepidopteran and dipteran species. In dipteran insects, the change of transcriptional profile and the crucial residue substitution of the lysozyme isoforms may drive the evolution of biological function into dual role: one role in a digestive manner to break down ingested bacteria in the gut, and the other role in a defensive response against pathogens that enter the haemocoel (Regel et al. 1998; Terra and Espinoza-Fuentes 1987; Bedoya et al. 2005). Possibly the diversity of genes and their functions in the dipteran and lepidopteran insects may be a consequence of the adaptation of insects to the environment: the bacteria-rich food source of dipteran insects may drive the evolution of the lysozyme multi-gene family and the dual digestive and immune functions.

When the sequences and structures of lysozymes were compared between lepidopteran insects and chicken, differences were noticed in the 3-D structure between chicken lysozyme and those lepidopteran lysozymes that have been reported to display antimicrobial activity against Gram-negative bacteria (Table 3 in Abraham et al. 1995; Yu et al. 2002). Two main differences of note were found. First, the spatial distribution of positively charged residues differs. In BmLZ and OstrinLysC, the distribution of positive charges has a tendency toward forming a positively charged side on the surface that may contact the negatively charged lipopolysaccharide in the bacterial cell wall (Figure 7 B-b, B-c; Matsuura et al. 2002). In contrast, the positive charges in HEWLZ are relatively dispersed (Figure 7 B-a; Matsuura et al. 2002). Second, the location of Loop-2 differs. In BmLZ and OstrinLysC, the shorter loop-2 (7 residues) contains two hydrophobic residues (proline and alanine). It locates in the positively charged-rich surface and is close to the cluster of hydrophobic residues (Figure 7B-b, B-c). In contrast, the location of the longer loop-2 (9 residues) of HEWLZ is in the upper portion of the molecule, and it is not close to the cluster of hydrophobic residues (Figure 7B-a). The longer loop-2 and its location change were also found in the digestive lysozyme of the housefly (Marana et al. 2006; Cancado et al. 2007). The hydrophobic outer membrane is believed to be a major barrier for most antimicrobial peptides that are not active against Gram-negative bacteria (Boman 2000; Papo et al., 2002; Papo and Shai 2003; 2005), such as most c-type lysozymes. The shorter loop-2 located close to the positively charged side and the cluster of hydrophobic residues might enable the lepidopteran lysozymes to overcome the hydrophobic outer membrane of Gram-negative bacteria. The structure change initiated by the molecular flexibility or plasticity, which is increased by loops and the proline residue, might affect the penetrability of lysozyme (Oh et al. 2000; Nagpal et al. 2002; Yang et al. 2006). Thus, we hypothesize that the positively charged-rich surface and the shorter loop-2 located close to the cluster of hydrophobic residues might be potential key factors for interaction with Gram-negative bacteria and incurring activity. To test this hypothesis, further investigation on the antibacterial spectrum of available lysozymes and modifying lysozymes molecules which changing the loop-2 and other crucial residues is ongoing.

In this decade, more lepidopteran c-type lysozymes have been identified. The information from the residue profiles and the conformation of the lepidopteran c-type lysozymes, which have antibacterial activity against Gram-negative bacteria, may shed light on the interaction between lysozyme and the cell wall of bacteria. The OstrinLysC and other lepidopteran lysozymes may be valuable as model molecules for understanding the mechanism of antibacterial peptides against Gram-negative bacteria. Furthermore, such molecules might be useful for designing new antimicrobial peptides for future therapeutic purposes.
